# Heavy Metal Poisoning and Cardiovascular Disease

**DOI:** 10.1155/2011/870125

**Published:** 2011-09-08

**Authors:** Eman M. Alissa, Gordon A. Ferns

**Affiliations:** ^1^Faculty of Medicine, King Abdul Aziz University, P.O. Box 12713, Jeddah 21483, Saudi Arabia; ^2^Institute for Science & Technology in Medicine, Faculty of Health, University of Keele, Staffordshire ST4 7QB, UK

## Abstract

Cardiovascular disease (CVD) is an increasing world health problem. Traditional risk factors fail to account for all deaths from CVD. It is mainly the environmental, dietary and lifestyle behavioral factors that are the control keys in the progress of this disease. The potential association between chronic heavy metal exposure, like arsenic, lead, cadmium, mercury, and CVD has been less well defined. The mechanism through which heavy metals act to increase cardiovascular risk factors may act still remains unknown, although impaired antioxidants metabolism and oxidative stress may play a role. However, the exact mechanism of CVD induced by heavy metals deserves further investigation either through animal experiments or through molecular and cellular studies. Furthermore, large-scale prospective studies with follow up on general populations using appropriate biomarkers and cardiovascular endpoints might be recommended to identify the factors that predispose to heavy metals toxicity in CVD. In this review, we will give a brief summary of heavy metals homeostasis, followed by a description of the available evidence for their link with CVD and the proposed mechanisms of action by which their toxic effects might be explained. Finally, suspected interactions between genetic, nutritional and environmental factors are discussed.

## 1. Introduction

The potential association between chronic heavy metal exposure and cardiovascular disease (CVD) has a number of implications. Although the cardiovascular system is not typically viewed as a primary target of heavy metal toxicity, review articles covering their role as cardiovascular toxicant are scant, and the prime concern of most reviews has focused on the imbalance in the antioxidant protective mechanisms leading to oxidative stress in the cells as a major effect of their environmental exposure. Altered gene expression by environmental influence, particularly dietary components over gene regulation is expected to be responsible for heavy metal toxicity.

In this paper, we will give a brief summary of heavy metals homeostasis, followed by a description of the available evidence for their link with CVD and the proposed mechanisms of action by which their toxic effects might be explained. Finally, suspected interactions between genetic, nutritional, and environmental factors are discussed.

## 2. The Prevalence of CVD and Its Risk Factors

Despite recent significant advances in the treatment of CVD, it remains the number one cause of death in the developed world and accounts for almost one million fatalities each year in United States alone [1]. CVD also accounts for 82% of deaths in the developing countries [2]. The annual mortality rate of CVD is expected to reach 23.6 million deaths by 2030 [3]. The traditional risk factors for CVD do not account for all deaths [4]. Environmental, dietary, and lifestyle factors appear to be important, accounting for the dramatic recent changes in prevalence and would be of wide public health significance. 

Confounding variables effects are being now evaluated as potential mediators (i.e., in the biological causal pathway), moderators (i.e., risk modifiers), direct causes, or otherwise parts of complex causal pathways [5]. These pathways can include connections between individual-level indicators (e.g., age, sex, race/ethnicity, socioeconomic status); behavioral risk factors (e.g., dietary habits); biological factors (e.g., genetics); social factors; heavy metals dose (i.e., both recent and cumulative); health conditions (e.g., diabetes, heart disease, and hypertension); other biological markers predictive of disease (e.g., homocysteine levels) that may be thought of as either outcomes by themselves or as intermediate pathological states that result in other conditions (e.g., renal dysfunction, cognitive declines).

The spectrum of risk factors for CVD ranges from purely genetic to behavioural and environmental factors in the broadest sense (Table 1). CVD is initiated by a coincidence of different risk factors. The latter two already show that behaviour and the environment (including the composition of nutrition) play an essential role in the majority of CVD. Patients differ in the time of onset, dynamics, and outcomes of CVD, indicating the complex pathophysiology of CVD. Different, genetically determined susceptibilities to environmental risk factors, interactions of the cardiovascular system with other organs like the immune system, and possible interactions between these risk factors within an individual are the likely causes of those differences. Despite an increasing understanding of genes, proteins, signalling pathways, cell-cell interactions, and systemic processes involved in CVD (initiation, progression, and outcome), the relevance of environmental factors is hardly investigated.

## 3. The Mechanisms of Atherogenesis

Atherogenesis is a multifactorial pathophysiological process of the arterial vasculature, which is characterized by progression from inflammation and smooth muscle cell proliferation to late stages that are marked by thrombotic and fibrotic obliterations of the vessels. Dysfunction of the endothelial cells leads to a series of events including inflammatory cell infiltration, platelet-thrombus formation, impaired nitric oxide (NO) homeostasis in the vessel and concomitant alteration of the cellular redox state [32]. Oxidized LDL particles are readily taken by macrophage scavenger receptors, leading to “foam cell” formation, that precedes atheroma development. Lipid aldehydes derived from LDL oxidation can also modulate expression of genes coding inflammatory mediators and adhesion molecules [33]. Reactive oxygen species can also function as signaling molecules that help to induce the activity of nuclear transcription factors such as nuclear factor Kappa B (NF-*κ*B). The increased activity of these transcription factors is associated with upregulation of vascular adhesion molecules-1 (VCAM-1), cytokines such as interleukin-1beta (IL-1*β*) and tumor necrosis factor alpha (TNF-*α*) and chemokines including monocyte chemoattractant protein (MCP)-1 and interleukin (IL)-8 in the endothelium [34]. Many risk factors, including cigarette smoking, hypertension, diabetes mellitus, and hypercholesterolemia, can induce atherogenesis by modulation of inflammatory potential, oxidative stress, or NO perturbations in the endothelium. 

There are several hypotheses to explain the initiation of CVD. Cumulative evidence from a large number of studies indicates that inflammation plays a pivotal role in atherosclerotic plaque formation [35]. Based on current knowledge, the hypothesis that best explains atherosclerosis pathophysiology is the “response to injury hypothesis” [34]. Lipid peroxidation is initiated by free radicals (e.g., superoxide anion, hydrogen peroxide, and lipid peroxide), which are produced in the body primarily as a result of aerobic metabolism [36, 37]. Transition metal ions, particularly divalent ions such as iron and copper, can further catalyze highly reactive free radicals formation in Fenton-type reactions [38]. LDL modification by oxidative damage is considered to be a key event in the development of atherosclerosis, and oxidized LDL particles are found in atherosclerotic lesions [33]. Although existing literature is limited, there are several mechanisms pointing to the atherogenic effects of heavy metals exposure by which they can promote lipid peroxidation and subsequent atherosclerosis.

## 4. The Toxic Effects of Heavy Metals

Heavy metals are commonly defined as those having a specific density of more than 5 g/cm^3^ such as lead, mercury, aluminum, arsenic, cadmium, nickel. They are widely distributed in the earth's crust, but present at very low concentrations in the body. Their presence in the atmosphere, soil, and water, even in traces, can cause serious problems to all organisms. Their main impact on human health is principally through occupational exposure, environmental contamination, and accumulation in food, mainly in vegetables grown on contaminated soil. Arsenic and cadmium, in addition to mercury and lead, have been identified as the most probable causes of heavy metal-related disease observed in primary care medicine [39]. Exposure to one heavy metal contaminant is often accompanied by exposure to others. It is, therefore, expected that joint interactions may occur in populations exposed to mixtures of metals. 

Heavy metals are toxic because they may have cumulative deleterious effects that can cause chronic degenerative changes [40], especially to the nervous system, liver, and kidneys, and, in some cases, they also have teratogenic and carcinogenic effects [41]. The mechanism of toxicity of some heavy metals still remains unknown, although enzymatic inhibition, impaired antioxidants metabolism, and oxidative stress may play a role. Heavy metals generate many of their adverse health effects through the formation of free radicals, resulting in DNA damage, lipid peroxidation, and depletion of protein sulfhydryls (e.g., glutathione) [42].

The importance of these metals as environmental health hazards is readily evident from the fact that they ranked in the top 10 on the current Agency for Toxic Substances and Disease Registry Priority List of Hazardous Substances [43]. This listing is based on the toxicity of the substance and the potential for exposure from air, water, or soil contamination. As a result of the extensive use of these metals and their compounds in industry and consumer products, these agents have been widely disseminated in the environment. Because metals are not biodegradable, they can persist in the environment and produce a variety of adverse effects. Maximum levels for heavy metals in food have been set in consideration for possible chemical contaminants. Although contaminated food may contain environmental toxins, they are also a very important source of nutrients, for example omega 3 fatty acids, which may prevent chronic diseases like CVD. Thus, an attempt has been made to allow people to obtain the beneficial health effects of natural food without excessive exposure to possible contaminants. Evaluations of heavy metals toxicity have been made by several international bodies, like the Center of Disease Control (CDC), World Health Organization (WHO), Occupational Safety and Health Administration (WHO-OSHA), International Programme on Chemical Safety (WHO-IPCS), Joint FAO/WHO Expert Committee on Food Additives (JECFA), and International Agency for Research on Cancer (IARC) (Table 2). Some of them have been classified as carcinogens of category 1 (cadmium). Currently, there are insufficient data to set a threshold value above which heavy metals would exert their negative effects. Defining this threshold might be fraught with difficulties since it might well be population-dependent due to differences in the population intake of dietary antioxidants or differences in their genetic-based defenses.

## 5. Potential Sources of Heavy Metals Contamination

The toxicity of heavy metals at high levels of exposure is well known, but a current concern is the possibility that continual exposure to relatively low levels of heavy metals may lead to chronic adverse health effects. Despite an overall decrease in human exposure to heavy metals in recent years, the potential for high intake of these contaminants still exists at many homes and in many occupational settings. Cosmetic products like lipsticks, eye makeup, Talcum powder, and skin lightening creams are potential sources of heavy metals exposure [44]. The presence of lead has been reported in traditional eye cosmetics such as Kohl and Surma [45]. Henna, a traditional plant product applied as temporary paint-on tattoos and hair dying, is reported to be very rich in heavy metals such as mercury and lead [46]. Other hidden sources may include ethnic folk remedies, toys, and certain imported candies and spices, [47–49]. Bottled Zamzam holy water, which is made available to pilgrims on sale, has been taken off the market recently for containing high levels of arsenic [50]. Tobacco plants have a special ability to absorb cadmium from soil and to accumulate it in the leaf [51]. Smoking of cigarettes and Shisha (hookah, narghile), a widely used smoking device in Saudi Arabia, is an important exposure route to cadmium [52].

## 6. The Metabolic Effects of Heavy Metals

The knowledge gained about the homeostasis of heavy metals has been substantial over more than a decade. Although they have no known metabolic function, when present in the body they disrupt normal cellular processes, leading to toxicity in a number of organs. They are relatively poorly absorbed into the body, but once absorbed are slowly excreted and accumulate in the body causing organ damage. Thus, their toxicity is in large part due to their accumulation in biological tissues, including food animals such as fish and cattle as well as humans. Distribution of heavy metals in the body relies on its binding to carrier molecules in the circulation. Metallothioneins are small proteins rich in cysteine residues, which accounts for the unique metal-binding properties of metallothioneins and play a major role in the dispersal and storage of heavy metals in the body. They also accumulate in hair and toenails (e.g., arsenic and mercury), which both can be used as indicators of long-term exposure in population studies. These heavy metals have a slow excretion rate from the body, as indicated by their long half-life time (e.g., half-life of lead is 27 year in cortical bone and 16 year in cancellous bone, half-life of cadmium is 10–30 years), compared with their uptake rate. 

### 6.1. Arsenic

After ingesting inorganic arsenic compounds, the absorbed arsenic is metabolized primarily by the liver and excreted by the kidneys into the urine within a few days after exposure. Organic arsenic species in fish are also rapidly absorbed. In comparison to inorganic forms, organic compounds are much less extensively metabolized in the human body and more rapidly eliminated in urine with less than 5% was found to be eliminated in feces. In addition to gastrointestinal, dermal, or pulmonary uptake, exposure to organic arsenic species originates from methylation of inorganic arsenic inside the human body, which is regarded as a detoxification mechanism, since the methylated metabolites exert less acute toxicity and reactivity with tissue constituents than inorganic arsenic. The central site for arsenic methylation in the human body is the liver. These methylated metabolites can be eliminated in the bile. Factors such as dose, age, gender, and smoking contribute only minimally to the large interindividual variation in arsenic methylation observed in humans (reviewed by [53]).

### 6.2. Lead

The gastrointestinal absorption of lead is higher for children (30–50%) than for adults (5–10%). The absorbed lead is distributed to blood, soft tissue, and bone. In blood, red blood cells virtually bind all of the lead (98-99%), thus only 1-2% of blood lead are present in plasma. Gastrointestinal absorption and retention, the major pathway of lead intake, have been shown to vary widely depending on the chemical environment of the gastrointestinal lumen, age, and iron stores (nutritional status of the subject). Certain dietary components may act by increasing lead solubility, such as ascorbic acid, amino acids, vitamin D, protein, fat, and lactose, thus enhancing its absorption. Total body content of lead does not have a feedback mechanism which limits its absorption. Absorbed lead is mainly excreted in urine, whereas the feces contain predominantly unabsorbed lead. Being one of the calcium-like elements, lead follows the movement of calcium in the body to a large extent, and physiologic regulators of calcium metabolism usually affect the behavior of lead in a similar manner. Although bone has been considered a storage site for more than 90% of the total body burden, increased bone turnover in times of physiological (e.g., pregnancy or lactation) and pathological (e.g., osteoporosis) conditions release lead from bone. Lead can be remobilized from bone by competing with calcium for transport and for binding sites and is released, along with calcium, when bone is resorbed (reviewed by [54]). The mechanisms by which both elements enter and leave the bone are similar and through these mechanisms, bone lead equilibrates with blood lead [55].

### 6.3. Cadmium

The possible range of intestinal absorption rate for cadmium was established to be between 3 and 7% in humans and was used to assign an average 5% absorption rate in deriving a safe exposure level [56]. However, higher cadmium absorption rates (20–40%) were observed among young subjects and considered biliary excretion and reuptake via enterohepatic circulation to be the most likely possible reason. The duodenal iron transporter is upregulated by iron deficiency, which leads to an increased intestinal absorption of dietary cadmium. This is probably the main reason why the body burden of cadmium is generally higher among women [57] whose prevalence of iron depletion is higher than that of men. Once absorbed, cadmium binds avidly to metallothionein. Cadmium irreversibly accumulates in the human body, particularly in kidneys and liver. Because there is no efficient excretory mechanism for cadmium from the body and it is bound with high affinity to metallothionein within cells. Accumulation of cadmium mainly in liver and kidney and also in testes is due to the ability of these tissues to synthesize metallothionein, a cadmium-inducible protein that protects the cell by tightly binding the toxic cadmium ion. The kidney is regarded as critical organ for its accumulation and toxicity. Greater than one-third of body cadmium deposits are found in the kidney, especially in subjects with low environmental exposure. By far, the most toxicological property of cadmium is its exceptionally long half-life in the human body and thus its low excretion rate (reviewed by [30]).

### 6.4. Mercury

Dietary methylmercury is well absorbed from the gastrointestinal tract, readily enters the bloodstream, and is distributed to all tissues. About 5% of the body load is found in the blood compartment, and about 10% is found in the brain. 95% of the methylmercury in blood is bound to erythrocytes leaving 5% present in plasma. Less than 1% of the body burden of methylmercury is excreted per day, mainly via the feces. In the body, methylmercury is mainly, if not exclusively, bound to the sulfur atom of thiol ligands. Methylmercury is metabolized to inorganic mercury prior to elimination via feces, but the rate of conversion is slow (the half-life is about 70–80 days). In the liver and kidney, it is rapidly converted to inorganic mercury and stored as divalent mercury cation. This, together with the fact that the human body has no way of excreting mercury actively, means that mercury continues to accumulate in the body throughout life (reviewed by [31]).

## 7. Health Harmful Effects of Heavy Metals

The severity of adverse health effects is related to the chemical form of heavy metals and is also time and dose dependent. As mentioned earlier, heavy metals as environmental pollutants and promoters of oxidative stress are associated with a multitude of disadvantageous impacts on human health. There is a growing concern about the physiological and behavioral effects of environmental heavy metals in human population. Human intoxication has both acute and chronic effects on health and environment (Table 2). Albeit the toxicity of heavy metals at high levels of exposure is well known, a major concern of today is the possibility that continual exposure to relatively low levels of heavy metals may entail adverse health effects. Nevertheless, their contribution to CVD is still incompletely understood. Recent studies have shown that vascular effects of heavy metals may contribute to a variety of pathologic conditions including diabetes mellitus and hypertension [58, 59]. Mechanisms of action after heavy metal intoxication are less well studied and are still unclear. 

### 7.1. Arsenic

Elemental arsenic is a metalloids found ubiquitously in nature. Humans are exposed to arsenic through medicinal, environmental, and occupational sources. Both organic and inorganic arsenic are present in various amounts in food-like marine fish. Organic forms are arsenobetaine, which account for 90% or more of the total arsenic in marine fish, and arsenocholine, in smaller amounts. However, inorganic forms of arsenic are much more toxic than the organic forms. Arsenic can exist in four valency states, trivalent (As^III^) and pentavalent (As^V^) arsenic are the major inorganic forms in natural water, whereas minor amounts of monomethylarsonic acid (MMA) and dimethylarsinic acid (DMA) can also be present. In the general population, the main exposure to inorganic arsenic is through ingestion of high-arsenic drinking water [60]. The safety level of arsenic in drinking water has been lowered from 50 to 10 ppb by the US Environmental Protection Agency [61].

Chronic arsenic intoxication seems to be an important public health problem in India, Bangladesh, Chile, Argentina, Hungary, Japan, and China [62]. Both environmental and occupational exposures to inorganic arsenic have been related to an increased cardiovascular mortality [63, 64]. Arsenic has been documented as the major risk factor of black foot disease, a unique peripheral vascular disease identified in endemic areas of arseniasis in Taiwan. However, other forms of peripheral vascular diseases have been shown to be caused by arsenic in other studies from several other countries. 

Clinical studies have also reported other arsenic-induced cardiovascular effects including hypertension, diabetes mellitus, atherosclerosis, coronary heart disease, and stroke in a dose-dependent manner [65, 66]. Previous reviews of the role of arsenic in CVD were supportive of the possibility of an association, but the evidence was inadequate to establish a causal-effect relationship. A causal inference may be established if the data had a stronger effect in a susceptible subgroup of the population. 

#### 7.1.1. Epidemiological Evidence

The studies on arsenic-induced CVD were either occupational cohort studies or ecological correlation studies. Although epidemiological studies conducted in general populations strongly support long-term arsenic exposure as an independent risk factor for CVD, the studies of occupational populations are inconclusive [67]. Methodological problems might limit the causal interpretation of this relationship. Occupational studies may be subject to biases resulting from the healthy worker effect, which may underestimate the arsenic-related risk due to the fact that the severely ill are ordinarily excluded from employment, and multiple exposure to various chemicals, and the correlation studies may have the problem of ecological fallacy. The limitation of nonoccupational studies includes the number of potential participants, accurate diagnosis of the cardiovascular endpoints, heterogeneity of exposure resources, limited exposure range which might be a challenge for epidemiological studies to have a valid long-term exposure measures, and interindividual variability to the cardiovascular effect of arsenic exposure.

Nonetheless the dose-response relationship and the biological plausibility for the association indicate that chronic arsenic poisoning is an independent risk factor for atherosclerosis [27]. Few epidemiologic studies in the US have reported the association of arsenic exposure with cardiovascular endpoints at low to moderate chronic levels in drinking water [68, 69]. Higher prevalence of ischemic heart diseases was found in subjects with cumulative arsenic exposure from drinking water, which was used as a marker of long-term exposure dosage, when compared to control subjects after multivariate adjustment [66]. Similarly, higher prevalence of hypertension was found among residents in endemic areas in Bangladesh of chronic arsenicism compared with those from nonendemic areas [70]. Inorganic arsenic exposure from drinking water, but not for the cumulative arsenic exposure, is also associated with an increased risk of developing type 2 diabetes mellitus [71]. Although hypertension and diabetes mellitus may partly explain the higher risk of CVD associated with arsenic exposure, the atherosclerotic effect of arsenic is independent because such an association persists even after controlling for the confounding effect of both factors. However, correcting for confounders in epidemiological studies is extremely challenging and is unlikely to completely account for their potential effects. An ecological study, conducted in the arseniasis-endemic areas of southwestern Taiwan, reported increased age-adjusted mortality from ischemic heart diseases compared with residents in nonendemic areas [64]. In Chile, acute myocardial infarction mortality increased following a period of high exposure to arsenic in drinking water and decreased after arsenic remediation had been implemented [72]. Likewise in southwestern Taiwan, mortality rates from ischemic heart disease were declining after the cessation of consumption of high arsenic well water [73]. However, results from studies conducted in endemic areas with chronic long-term arsenic exposure may limit the applicability to other populations, especially those with lower levels of arsenic exposure.

Except for a few studies using a prospective follow-up design, most are observational and cross-sectional. However, most of the existing epidemiological studies were conducted in populations with high levels of arsenic exposure, and little is known about the associations between chronic low level arsenic exposure via drinking water and CVD [74]. Furthermore, the heterogeneity of drinking water resources and the limited exposure range together pose a challenge for epidemiological studies to be conducted in other areas with low to moderate arsenic exposure levels that are relevant for most parts of the world and to have valid long-term arsenic exposure measures at the individual level.

#### 7.1.2. Mechanism of Action

One of the suggested mechanisms by which arsenic exerts its toxic effect is through an impairment of cellular respiration by inhibition of several carbohydrates enzymes (i.e., gluconeogenesis and glycolysis pathways) and the uncoupling of oxidative phosphorylation [75]. This might explain the link between acute arsenic exposure and diabetic risk through its influence on the expression of gene transcription factors that are related to insulin pathways, such as, insulin upstream factor 1 (IUF-1) in pancreatic cells or peroxisome proliferative-activated receptor *γ* (PPAR*γ*) in preadipocytes. Arsenic could also influence diabetes development by other mechanisms, including oxidative stress, inflammation, or apoptosis, nonspecific mechanisms that have been implicated in the pathogenesis of type 2 diabetes [76]. Future research should evaluate whether these mechanisms mediate the role of arsenic in diabetes development.

Other studies have suggested the involvement of oxidative stress in the pathogenic effects of arsenic exposure [77]. Available data suggest an important arsenic role based on a range of effects related to oxidative stress and vascular inflammation. Findings from mechanistic studies in animal/experimental studies suggest that arsenic causes inflammation in vascular tissues and activates oxidative signaling. The expression of chemokines and proinflammatory cytokines like monocyte MCP-1 and IL-6 has been induced in vitro by sodium arsenite in vascular lesions [78]. These observations are consistent with studies illustrating increased expression of circulating lymphocyte MCP-1 mRNA and plasma MCP-1 concentration in humans exposed to arsenic [79]. A more recent study also shows that occurrence of carotid atherosclerosis among subjects with genotypes of ApoE and MCP1 when exposed to high arsenic in drinking water [80].

Experimental studies have suggested that arsenic increases the production of reactive oxygen species [81]. Increased accumulation of arsenic in the vessel wall and increased atherosclerotic lesion formation were observed in the aorta of female ApoE-knockout mice given drinking water containing high concentrations of sodium arsenite without increasing plasma cholesterol. Characterization of these lesions illustrated increased macrophage accumulation and fibrosis in arsenic-exposed mice as compared to water-fed controls [82]. However, very little is known about the biochemical mechanisms by which low levels of arsenic exerts its proatherogenic effects.

Oxidative stress has been implicated in the pathophysiology of atherosclerosis [34]. The inflammatory process may be involved in the arsenic-induced atherosclerosis as shown by positive association between blood arsenic with plasma level of reactive oxidants (superoxide (O_2_^–^) and hydrogen peroxide (H_2_O_2_)) and by a negative association with antioxidant capacity [83]. Arsenic-induced oxidants, superoxide, and hydrogen peroxide have been implicated in in vitro studies [77]. Several cytokines and growth factors involving inflammation were upregulated in persons with an increased arsenic exposure [79]. In individuals with arsenic-related skin lesions in Bangladesh, plasma levels of systemic inflammation and endothelial dysfunction markers (such as sICAM-1 and sVCAM-1) were positively associated with serum arsenic concentrations [84]. Markers of systemic inflammation and endothelial dysfunction were found to be predictive for CVD [85]. Thus, suggesting a possible mechanism through which long-term arsenic exposure may affect CVD development. Thus the biomarkers of early biological effects of ingested inorganic arsenic may include blood levels of reactive oxidants and antioxidant capacity, inflammatory molecules, as well as cytogenetic changes.

Oxidized lipids are present in all stages of atherogenesis, and they generate several bioactive molecules (e.g., peroxides and isoprostanes), of which aldehydes (Malondialdehyde and 4-hydroxy-trans-2-nonenal) are the major end products [86]. Increased plasma levels of free lipid aldehydes and increased accumulation of their protein adducts in atherosclerotic lesions were detected in experimental animals [87]. Since lipid aldehydes are highly reactive and can increase monocyte adhesion, cytokine production, and lipid uptake by scavenger receptors, it is conceivable that excessive generation of these aldehydes or decreased detoxification upon arsenic exposure exacerbates atherosclerotic lesion formation [88].

#### 7.1.3. Combination of Gene-Environmental-Nutrient Interactions

Arteriosclerosis can occur following chronic arsenic poisoning irrespective of traditional coronary risk factors [89]. However, one would not expect every arsenic-exposed individual to develop CVD. This implies that other factors might affect the development and progression of arsenic-induced CVD. The epidemiological literature to date suggests that the cardiovascular effects of arsenic exposure are modified by nutritional factors, genetics, and arsenic metabolism capacity. These studies have clinical implications on the management and prevention of arsenic-induced CVD. It is known that both genetic and acquired susceptibility may modify the risk of arsenic-induced CVD [90].

Plausible mechanisms for the effect of arsenic on CVD include oxidative stress as previously explained, antioxidant enzymatic inhibition such as glutathione reductase, glutathione S-transferase, and glutathione peroxidase, and altered gene regulation, which might be implicated in the endogenous defense against arsenic's effect. Glutathione S transferases (GSTs) are a superfamily of enzymes that is important for the detoxification reactions in xenobiotic metabolism and plays a major role in cellular antioxidant defense mechanisms [91]. Glutathione has also been suggested to be a necessary component for arsenic metabolism probably in the initial reduction of arsenate to arsenite and in subsequent oxidative methylation. In a large study conducted in northeastern Taiwan with low-to-moderate exposure, the prevalence of carotid atherosclerosis was significantly associated with the genetic polymorphism of GST; P1 and p53 [92]. The induction of oxidative stress by arsenic may influence gene expression, inflammatory responses, and endothelial NO homeostasis [93], which play an important role in maintaining vascular tone [94]. A causal relationship was suspected in a study on human gene expression related to arsenic-associated atherosclerosis among residents of endemic areas in Taiwan. Significant differences in gene expression, encoding for several cytokines and growth factors involving inflammation such as IL-1*β*, IL-6, and matrix metalloproteinase 1, were found among groups with varying prolonged exposure levels to arsenic [79]. In a small study in residents of a high-exposed area in Taiwan, genes encoding for antioxidant enzymes, like NOS3, the gene for endothelial nitric oxide synthase; SOD2, the gene for manganese superoxide dismutase, and CYBA, the gene for p22 phox [a critical enzyme for superoxide production and an essential component of nicotinamide adenine dinucleotide phosphate (NADPH) oxidase (NOX)], were found to be significantly associated with hypertension risk [95].

Several nutritional factors were investigated in relation to arsenic related cardiovascular effects. Selenium is a nutritionally trace element that has been known as an antagonist of arsenic toxicity [96]. Being also protective against oxidative stress, selenium supplementation was found to be effective in treating arsenism, an endemic chronic arsenic poisoning condition in China [97]. Consistent with this, some dietary deficiencies were found to interact with arsenic. For example, poor dietary selenium and zinc have been suggested as an underlying factor for arsenic toxicity in Taiwan and Bangladesh, well-known regions for their reduced selenium and zinc status worldwide [98].

Low serum carotene level has been suspected to increase the susceptibility to cardiovascular effects of arsenic exposure among residents of southwestern Taiwan villages with chronic arsenic exposure [99]. However, the potential mechanisms involved in the protective action remain to be studied. In the Health Effects of Arsenic Longitudinal Study (HEALS) in Bangladesh, the effect of low-level arsenic exposure on blood pressure was found to be highly correlated and was more pronounced in persons with lower intake of other micronutrients with known antioxidant action [100]. Arsenic exposure in the presence of inadequate intake levels of B vitamins and folic acid may affect blood pressure through its effect on the formation of S-adenosylhomocysteine and homocysteine. Historically, methylation of arsenic has been regarded as a detoxification pathway that takes place in the liver [101] and requires the conversion of S-adenosylmethionine to S-adenosylhomocysteine, which subsequently forms homocysteine, which requires sufficient levels of vitamin B2, B12, B6, and folic acid in body to be metabolized. Hyperhomocysteinemia, a novel cardiovascular risk factor [102], has been associated with high blood pressure [103]. Hence arsenic may contribute to the increase of homocysteine levels by consuming the S-adenosylmethionine pool and therefore enhance the subsequent cardiovascular risk.

Nevertheless, it is conceivable to presume that the findings from Taiwan and Bangladesh may not be generalizable to other populations due to several potential reasons like variations in the distribution of polymorphisms in genes involved in arsenic metabolism or response [104], differences in arsenic species to which populations were exposed or other coexposures [105].

### 7.2. Lead

Lead exposure assessments have been based on its intake from food, water, or air. Possible routes for lead exposure are inhalation and swallowing. The four main sources of contamination of food are soil, industrial pollution, agricultural technology, and food processing. Worldwide, there are six sources that account for most cases of lead exposure: gasoline additives, food-can soldering, lead-based paints, ceramic glazes, drinking water pipe systems, and folk remedies [106]. Depending on the source, the concentration, and the bioavailability of lead determined by the physical and chemical form of lead, the relative contribution of each source may vary considerably. Although important measures have been implemented in a number of countries to decrease environmental lead exposure such as the use of unleaded gasoline, removal of lead from paint, solder of canned foods, and glazed ceramics used for storage and preparation of food, it is still a major environmental health problem in specific communities and targeted high-risk populations. 

Even though safety standards of the WHO-OSHA for blood lead in workers have been established at 40 *μ*g/dL, no safe level of lead exposure has yet been defined, as health risks associated with lead are found at ever lower doses. The CDC statement concerning lead poisoning in young children redefined elevated blood lead levels as that ≥10 *μ*g/dL and recommended a new set of guidelines for treatment of lead levels ≥15 *μ*g/dL [107]. However, it has been suggested that the criterion for elevated blood levels in children is too high in adults based on substantial evidence [108]. 

There is a great public health concern in the effects of environmental lead exposure on cardiovascular outcomes [109], especially the role of chronic low-level lead exposures in the pathogenesis of CVD [110]. Population-based studies on the cardiovascular effects of lead have focused largely on the association with hypertension, a leading risk factor for CVD morbidity and mortality [111]. The interrelationship between blood lead and blood pressure has been reviewed and reported to be statistically significant [112]. However, a major drawback of this meta-analysis was the inclusion of lead exposed subjects with occupational and non-occupational sources. In as much other cardiovascular events including, coronary heart disease, stroke, and peripheral arterial disease, were found to be associated with lead exposure, the exact role of lead in CVD is still incompletely understood [25]. 

#### 7.2.1. Epidemiological Evidence

Lead intoxication has been shown to promote atherosclerosis in experimental animals [113]. Experimental findings in several species suggest that lead acts at multiple sites within the cardiovascular system [114]. Depending on the magnitude and the duration of lead exposure, cardiac and vascular complications are potentially life threatening. There are also indications that chronic lead exposure may affect systemic lipid metabolism [113]. Current evidence on lead-induced oxidative stress has been based mostly on in vitro experiments [115] or studies conducted in animals [116]. Chronic exposure has been also linked to atherosclerosis and increased cardiovascular mortality in man [117]. Several epidemiological studies among workers with high occupational exposure to lead have reported associations between lead exposure and oxidative stress markers [118]. Recent epidemiological studies have reported that low level lead exposure has a graded association with several disease outcomes such as hypertension and peripheral artery disease [119–121]. Although such diseases include components of oxidative stress, the relevance of oxidative stress to lead-related disease with low-level exposure has been criticized because mechanistic studies have been conducted at levels not typically observed in general population. The association between blood lead level and elevated blood pressure is still subject to controversy. However, lead has been postulated as causing hypertension by inducing an alpha adrenoceptor-mediated vasoconstriction [122]. Increased renin and angiotensin production, due to the nephrotoxicity of lead, could also be a factor in causing elevated blood pressure [123]. Lead-mediated impaired vasomotor tone, as a result of reduced NO bioavailability, may contribute to hypertension and hence atherosclerosis. Disturbances in calcium metabolism, particularly its role in modulating blood pressure through control of vascular tone, have been suspected as the likely mechanism of action. Moreover, cumulative evidence from clinical studies on the association between blood lead levels and CVD has yielded conflicting results [120, 124, 125]. Lack of consistency in findings could be due to differences among study cohorts in exposure/ toxicokinetic factors (e.g., dose, timing), in pattern of environmental characteristics (e.g., coexposures, comorbidity, developmental supports, assessment setting), in distribution of genetic characteristics that affect lead metabolism and racial background or the health worker effect [126, 127]. Methodological limitations are a great threat to validity of epidemiological studies, for example, misclassification of exposure and/or outcome may have occurred and resulted in further underestimation of the association of lead and cardiovascular end points.

Therefore, the results of these studies should be considered within the context of its possible limitations such as, the reliance on a single lead measurement, the use of different exposure measures, or residual confounding by sociodemographic determinants of lead exposure. The healthy worker effect may also lead to underestimate or invalidate the risk assessment of CVD.

#### 7.2.2. Mechanism of Action

Acute lead exposure has been reported to affect cardiac function, and chronic lead exposure has been shown to affect the electrical and mechanical activity of the heart and to alter vascular smooth muscle function in experimental animals [128]. Many studies have focused on metal-induced toxicity and carcinogenicity, emphasizing their role in the generation of reactive oxygen and nitrogen species in biological systems. Metal-mediated formation of free radicals may enhance lipid peroxidation and changes in calcium and sulfhydryl homeostasis. By promoting reactive oxygen species production, lead may trigger a cycle of oxidative stress and inflammation in the target tissues [129]. Depletion of cells' major sulfhydryl reserves seems to be an important indirect mechanism for oxidative stress that is induced by redox-inactive metals [130].

The precise mechanism explaining the hypertensive effect of lead exposure is unknown. However, an inverse association between estimated glomerular filtration rate and blood lead levels below 5 *μ*g/dL has been observed in general population studies [108], indicating that lead-induced reductions in renal function could play a major role in hypertension. Other potential mechanisms include enhanced oxidative stress in the pathogenesis of lead-induced hypertension [131]. Although elevated blood pressure and impaired renal function are proposed mechanisms that mediate the effects of lead on clinical cardiovascular outcomes, other mechanisms are likely to be involved.

As has been mentioned earlier, CVD progression and outcomes rely to some degree on the presence of inflammation [34]. Increased expression and production of inflammatory markers in association with lead exposure have also been found in humans [132]. Although these findings suggest a possible involvement of oxidative stress in the pathophysiology of lead toxicity, it is not clear whether these alterations are the cause of the oxidative damage or a consequence of it.

Various in vitro and in vivo studies have explored the underlying mechanisms by which chronic low level lead exposure can raise arterial pressure, thereby CVD development. These studies have identified the involvement of oxidative stress and inflammation [133], by promoting endothelial dysfunction [134], promoting vascular smooth muscle cells proliferation and transformation [135], and impairing NO homeostasis [136]. NO plays multiple physiological roles in vascular wall including endothelium-mediated vasodilatation, inhibition of platelet activation and smooth muscle cell migration and proliferation, and suppression of the proinflammatory mediators through NF-*κ*B inactivation [32]. Diminished NO bioavailability may be caused by inhibition of the endothelial NO synthase (eNOS) expression, a lack of substrate or cofactors for eNOS, alterations of cellular signaling such that eNOS is not appropriately activated, and, finally, NO inactivation through interaction with reactive oxygen species (O_2_^–^). Furthermore, antioxidant therapy with vitamin E and ascorbic acid supplementation raised NO availability in rats with lead-induced hypertension compared with no effect upon blood pressure or tissue nitrotyrosine (a marker of NO oxidation) in control rats [137]. These findings support the notion that exposure to lead causes functional NO deficiency, in part by reactive oxygen species mediated NO inactivation. Given the critical role of NF*κ*B in many aspects of atherogenesis, their activation by lead exposure may play a part in the development of hypertension [138].

Experimental studies have suggested plausible mechanisms whereby lead contributes to alterations of vascular resistance and hypertension by causing disturbances in calcium metabolism, particularly its role in modulating blood pressure through control of vascular tone [139]. It has been shown that lead can compete with calcium for the transport by channels and pumps involved in movements of ions across the cell membrane and between cytoplasm, endoplasmic reticulum, and mitochondria, thereby contributing to the changes in cytosolic calcium ions known to be involved in the regulation of vascular tone and vascular smooth muscle contraction [140]. In addition, lead may affect the calcium-mediated control of vascular smooth muscle contraction via serving as a substitute for calcium in calcium-dependent signaling pathways by interacting with calmodulin and calcium-dependent potassium channels [141]. Uncontrolled release of calcium ions from the mitochondria has been reported to occur during oxidative stress, a condition resulting from the imbalance between the production of free radicals and the counteraction by the cellular antioxidant defenses [142]. Lead may also increase pressor responsiveness to catecholamines, which may be a consequence of the lead effect on the intercellular messenger protein kinase C and its role in smooth muscle contraction [143].

#### 7.2.3. Combination of Gene-Environmental-Nutrient Interactions

The exact mechanism by which lead induces oxidative stress is not fully understood. However, at least certain circumstances (i.e.) including genetic predisposition, nutritional influence, and environmental coexposure are expected to interact and therefore are linked in an attempt to explain such mechanism(s) of lead-induced toxicity.

Nutrition is an important susceptibility factor suggesting that people with poor nutrition are particularly susceptible [144]. Nutritional factors are often considered as important modifier of the metabolism and toxicity of lead [145]. This can be explained by lead-induced oxidative stress, an effect that is augmented by lead-induced inhibition of several of the antioxidant systems. This supposition was confirmed by studies which showed extensive accumulation of reactive oxygen species (as markers of NO oxidation) in kidney, brain, and cardiovascular tissues of untreated rats with lead-induced hypertension and its reversal by antioxidant therapy using high doses of vitamin E and vitamin C [137]. Essential elements, such as calcium, zinc, iron, selenium, and antioxidant vitamins have shown to counteract the toxic effects of lead [146]. The joint effect of high lead and low antioxidant micronutrients levels should be considered as a modifying factor in atherosclerosis, and their role in determining risk should be investigated. These nutritional facts suggest a novel approach to strategies for treating environmental lead toxicity with micronutrients supplementations.

Certain genetic polymorphisms can lead to differences in the level of susceptibility to adverse effects of lead environmental exposure. A better understanding of the genetic factors that influence susceptibility to lead-induced intoxication could have significant importance for public health and intervention initiatives [147]. It is therefore reasonable to conjecture that genetic disposition could lead to differences in susceptibility to lead poisoning among the human population. Researchers have identified a small number of genes that induce susceptibility to environmental toxicants, and much interest has developed in that area. Three polymorphic genes have been identified that can influence the bioaccumulation and toxicokinetics of lead in humans, the 6-aminolevulinic acid dehydratase (ALAD) gene, the vitamin D receptor (VDR) gene, and the hemochromatosis (HFE) gene. 

One of the most important mechanisms of lead toxicity is its inhibitory effect on the heme biosynthetic pathway enzymes; ALAD and ferrochelatase. Polymorphisms of the ALAD gene have been associated with the accumulation and distribution of lead in the blood, bone, and internal organs. Lead binds the enzyme's sulfhydryl group, which normally binds zinc, preventing the binding of aminolevulinic acid (ALA), the normal substrate. ALAD activity has been used as a sensitive marker for the detection of lead intoxication [148]. Concomitantly, ALA accumulates in blood and urine and may contribute to lead-induced toxicity to the brain. Its urinary excretory level has been used as a biomarker for early lead exposure [149].

1*α*25(0H)_2_D_3_ (calcitriol), the circulatory form of vitamin D in blood, is involved in calcium absorption. It binds to VDRs in gut, kidneys, and bone. The VDR gene has been implicated in the control of calcitriol levels in serum, which normally regulates calcium absorption and can in turn affect lead levels. The high-affinity VDR appears to activate genes that encode calcium-binding proteins such as calbindin-D, which is involved in intestinal calcium transport. Because of their similar biochemical nature as divalent cations, calcium and lead often affects the normal function of calcium-dependent systems [150]. These data suggest that calcium and lead are cotransported through the gut into the blood, and from there the two metals may be codistributed to calcium-rich tissues such as the bone [151]. In addition, lead toxicity may impair calcitriol hormonal synthesis in the kidney, therefore interfering with calcium absorption [152]. Together, these data show that the interactions between lead, calcium, and calcitriol are complex and induce modifications of mineral and vitamin levels.

Hemochromatosis is the genetic form of iron load in which patients lack a functional HFE protein involved in iron homeostasis due to mutations in the HFE gene that may also influence lead absorption [153]. At least two mechanisms for the increased absorption of lead in hemochromatosis gene carriers have been postulated. HFE protein binds to the transferrin receptor, reducing its ability to bind to transferrin and thus decreasing the absorption of iron in the gut [154]. Consistent with us, an important association has been made between iron deficiency and increased lead absorption and hence toxicity [144]. HFE protein may also influence the expression of other metal transporters such as divalent metal transporter in the gut that modify the absorption of other metals in addition to iron [155]. This is compelling data that iron status influences lead toxicity. 

Therefore, differences in the expression rate of the polymorphic genes in response to nutritional influence over their activities are highly suggestive but not conclusive in considering environmental link with both genetic and dietary elements.

### 7.3. Cadmium

Cadmium is a widespread toxic metal contaminating many areas, either naturally or because of industrial use as in regions of Belgium, Sweden, UK, Japan, and China. Modes of exposure are either through intake of contaminated food (e.g., leafy vegetables, grains, organ meats, and crustaceans), drinking water, or by inhalation of polluted air or occupational in industries. Cadmium presence in tobacco smoke further contributes to human exposure as the tobacco leaves accumulate cadmium in a manner similar to certain food from plants. Smokers have approximately twice the cadmium body burden of nonsmokers [156]. 

Regardless of the route of exposure, cadmium is efficiently retained in the organism and remains accumulated throughout life. In addition to its cumulative properties, cadmium is also a highly toxic metal that can disrupt a number of biological systems, usually at doses that are much lower than most toxic metals. A European risk assessment report proposed that cadmium deleterious effects may occur at levels as low as 0.5 *μ*g/g creatinine based on data from the most recent European studies [157]. 

A threshold value for safe dietary cadmium exposure level has been set to be below 2.5 *μ*g/kg body weight per week [158]. Furthermore, it was noticed that subgroups of the population, such as vegetarians, women in reproductive phase of life, smokers, and people living in highly contaminated areas may exceed the tolerably weekly intake by about 2-fold.

Chronic cadmium exposure is associated with hypertension and diabetes [24, 159]. However, the exact influence of cadmium on the cardiovascular system remains controversial. More importantly, these data show that cadmium may exert effects on the cardiovascular system at extremely low exposure levels. In vitro studies data revealed that low-dose cadmium levels (well below toxic concentrations) may contribute to the initiation of pathophysiological changes in the vessel wall [160].

Several important reviews have outlined the cardiovascular effects of cadmium in man. Evidence from prospective studies reveals potential causal relationships of blood cadmium and blood pressure but not the relationship between urinary cadmium and hypertension [161]. An inverse relationship between urinary cadmium levels and blood pressure was reported in another meta-analysis [24]. These paradoxical relationships were evident in both high- and low-exposure populations and thus contradict earlier assumptions that this inverse association only reflected higher cadmium exposures. A limitation common to all these studies and thus to this meta-analysis is that the outcome was not consistently defined across studies; therefore, lack of association might reflect outcome misclassification. 

#### 7.3.1. Epidemiological Evidence

The cardiovascular effects of cadmium have been observed in in vitro studies and in experimental animal models [162, 163]. Increased cardiovascular mortality was documented for men living in areas with increased potential for cadmium exposure, thus suggesting that cadmium is at least a comorbidity factor if not a causative factor [57].

Epidemiologic studies of the association of environmental cadmium exposure with blood pressure end points are inconsistent. Discrepancies across epidemiological studies might be due to that some studies have strengths including prospective designs [164], blood pressure values entry as a continuous variable to avoid outcome misclassification bias [165], while other studies, however, have been limited by small sample sizes, lack of adjustment for potential confounders, and lack of standardization of blood pressure measurements [161]. Sample selection considerations and exposure measurement error are additional limitations in these studies [166]. 

Cadmium exposure also potentiates some diabetic complications related to renal tubular and glomerular function. Epidemiological evidence shows higher susceptibility for persons with diabetes to develop cadmium induced renal dysfunction [167]. A study examining the data from National Health and Nutrition Examination Surveys (NHANES) reported a significant association between high urinary cadmium levels and high fasting blood glucose levels in a dose dependent manner, as well as more susceptibility among the diabetic subjects for the cadmium-induced renal effects [168]. Thus, suggesting that cadmium may be a cause of prediabetes and diabetes mellitus in humans. On the contrary, less agreement exists about the clinical significance and predictivity of the urinary cadmium level as a surrogate marker of body content and the tubular biomarkers of renal dysfunction.

NHANES data reported that peripheral arterial disease might be associated with blood and urinary cadmium, thus suggesting that cadmium is involved in arterial dysfunction [58]. Different cadmium biomarkers may provide different information regarding the timing and source of exposure. However, the use of these biomarkers has been inconsistent across epidemiological studies. In general, urinary cadmium level reflects the body burden over long-term exposure among people with lower, nonoccupational exposures, and blood cadmium, with a half-life of 3-4 months, is considered an indicator of recent exposure [56]. Alternatively, urine and blood cadmium are sometimes considered biomarkers of ongoing and long-term cadmium exposure, respectively [156]. 

Cadmium may exert its adverse cardiovascular effects by promoting atherosclerosis and by inducing disadvantageous cardiac functional and metabolic changes [169]. Recently blood cadmium level was independently associated with myocardial infarction [170] and early atherosclerotic vessel wall thickening as estimated by intimamedia thickness ratio [171]. In contrast no correlation was observed between blood cadmium and measures of arterial function [172]. Epidemiological studies did not firmly establish a link between cadmium and CVD due to confounding effects, for example, coexposure to other heavy metals, unadjusting for smoking habits. Moreover, disagreement between exposure studies might be attributed to the use of different exposure measures with different pathophysiological significance of blood and urinary cadmium.

#### 7.3.2. Mechanism of Action

It has been long hypothesized that cadmium may contribute to the pathogenesis of CVD via a number of proposed mechanisms, such as partial agonism for calcium channels, direct vasoconstrictor action, and inhibition of vasodilator substances such as NO [173]. The exact mechanism whereby cadmium affects the cardiovascular system is not known, although experimental studies have suggested several plausible possibilities [174]. Because cadmium levels used in experimental models are much higher than exposure in the general population, the relevance of these mechanisms to the pathogenesis of CVD is uncertain. A primary mechanism for cadmium toxicity is its effect on cells which has been ascribed to the oxidative stress promoting cadmium action, as observed in vivo [162], and most importantly, the depletion of glutathione and alteration of sulfhydryl homeostasis [42], thus indirectly increasing oxidative stress and lipid peroxidation [175]. However, results from other studies were inconclusive in supporting a direct effect of cadmium [171]. It has been argued that reasons are the concentration of cadmium applied, as well as the upregulation of antioxidant defense in endothelial cells in response to cadmium may define the presence of reactive oxygen species in endothelial cells [176].

Cadmium is absorbed mainly through the respiratory and digestive tracts and under conditions of chronic exposure; cadmium is transported in blood bounded mainly to metallothionein. Metallothioneins are heavy metal-binding proteins that can protect against heavy metal toxicity and oxidative stress. The vascular wall has been shown to be a target organ of cadmium deposition [177]. However, other important issues are not yet fully understood, like the form of cadmium which is taken up by cells (i.e., free ion or protein bound), their expected amounts in circulation, and the precise uptake route of cadmium by the cells. Albeit, several ion channels and transporters have been described to transport cadmium across the plasma membrane, for example, calcium-channels [178], plasma membrane-associated DMT-1 [179]; it is unclear whether these mechanisms are also active in endothelial cells. 

Apart from direct uptake of cadmium by endocytosis into cells of the vessel wall, cadmium may also be taken up by cells of the immune system and may enter the vessel wall via infiltration of the vessel wall, for example, by cadmium-laden monocytes [180]. Given the fact that the critical role of monocytes/macrophages transdifferentiate into foam cells and necrotic foam cell death in many aspects of endothelial dysfunction, their excessive production by cadmium plays a major part in the initiation and promotion of atherosclerosis. Cadmium uptake could also occur via disruption of endothelial integrity and subsequent cadmium-mediated endothelial cells death. Formation of gaps between endothelial cells usually follows, allowing for cadmium diffusion from the blood stream into the medial layer [59]. Vascular wall cells seem to allow for a sufficient transport of cadmium across the endothelium and are capable of retaining high amounts of cadmium mainly in the smooth muscle cells [177]. Effects on smooth muscle cells include an interaction with ion homeostasis and Ca^2+^ flux, cytotoxic effects, but also the stimulation of smooth muscle cell proliferation at low cadmium concentrations [181], thus, allowing for subsequent lipid accumulation in the vessel wall and a modification of lipid profiles towards a more atherogenic state [34]. Ample of evidence pinpoints the induction of endothelial cell death by cadmium which is thought to be fundamental in the atherosclerosis-promoting properties of cadmium [59]. However, data on the mode of cell death are contradictory. Cadmium-induced endothelial necrosis would, in addition to damaging the integrity of the vascular endothelium, also contribute to vascular inflammation.

#### 7.3.3. Combination of Gene-Environmental-Nutrient Interactions

There is evidence for nutritional influence over the rate of intestinal absorption of cadmium (i.e.) increased cadmium if the nutritional intake of calcium, iron, or zinc is low [182]. Moreover, cadmium exposure interferes with the homeostasis of other metals, and, reciprocally, cadmium effects depend on the body status for some essential metals [183]. Cadmium is acquired by transport mechanisms developed for essential metals, most likely to be one of the following divalent cations: zinc (Zn^2+^), iron (Fe^2+^), manganese (Mn^2+^), and calcium (Ca^2+^). It follows that the mechanisms of cadmium toxicity must be considered with respect to the systems regulating different aspects of these metals turnover in the body. Considering that cadmium substitutions at the metal sites of metalloproteins were performed in vitro only and the scarcity of data demonstrating such occurrences in vivo, care should be taken before interpreting cadmium toxicity data with a simple molecular explanation. Yet, cadmium replacement of other metals in cellular proteins does occur as in metallothionein [184].

Furthermore, metallothionein may, apart from binding and thereby inactivating the major portion of cadmium ions, also serve as a source for constant levels of intracellular free cadmium ions [59]. On the other hand, recent epidemiological studies are indicating the protective effect of the antioxidant property of zinc against cadmium toxicity probably by metallothionein stimulation [171]. To date, mechanisms of cadmium-zinc interaction and their impact on the oxidative status derived from in vitro studies and limited number of human studies [185]. The wide variety of different doses, dose ratios, element administration modes, and exposure lengths of cadmium and zinc often yielded contradictory results. 

Cadmium intoxication results in an induction of metallothionein gene transcription and an increase in metallothionein production [186]. Cadmium also affects several genes involved in the stress response to pollutants or toxic agents, as in heat shock proteins that are highly implicated in cardiovascular pathophysiology [187]. Many genes involved in cell cycle regulation are overexpressed after exposure to cadmium, and many proteins are upregulated; for example cadmium stimulates the expression of ICAM-1 [188].

Cadmium affects cell cycle progression, proliferation, differentiation, DNA replication, and repair as well as apoptotic pathways [147, 158]. In addition to its role as a generator of reactive oxygen species, involved in the occurrence of DNA damage, cadmium may also reduce cellular antioxidants levels [189]. The reduction of activities of several antioxidant proteins (catalase, glutathione reductase, total glutathione), mediated by cadmium, may cause the accumulation of reactive oxygen species in cells [190]. Indirectly, this overproduction of oxidant molecules may be also responsible for the generation of abnormal or misfolded proteins and lipid peroxidation [191].

### 7.4. Mercury

Mercury is an environmental pollutant that presents at low levels in water systems (lakes, rivers, oceans, etc.) but bioconcentrate in the aquatic food chain, as in some fish species (particularly fatty fish) that can also contain other environmental contaminants such as polychlorinated biphenyls, dioxins. The global cycle of mercury begins with the evaporation of mercury vapor into the atmosphere. More concern about the release of volatile mercury that will become part of the local mercury cycle and repollute the environment again, into the ambient air. Mercury exists in three forms: elemental or metallic mercury, inorganic mercury compounds, and organic mercury. It is used in glass thermometers as elemental mercury and in dental amalgam fillings as inorganic mercury compounds. Organic mercury is found mainly in fish as methylmercury and in some vaccines as ethylmercury (thimerosal). 

The US Environmental Protection Agency has reduced the recommended safe daily intakes of methylmercury from 0.5 to 0.1 *μ*g/kg body weight [192]. In the absence of advisories for local waters which are available, US Environmental Protection Agency and US Food and Drug Administration have also issued recommendations on fish consumption among women of childbearing age and young children based on methylmercury content; commonly eaten fish and shellfish that have lower levels of mercury should be limited to two meals per week.

In recent years, more attention has been given to other health effects of methylmercury exposure, following the epidemiological findings from Finland, confirming that high mercury content in hair was associated with an increased progression of atherosclerosis and risk of CVD [193]. It is noteworthy that these adverse effects on CVD have been observed at methylmercury levels much lower than those associated with neurotoxicity. 

#### 7.4.1. Epidemiological Evidence

Mercury exposure has been shown to promote atherosclerosis both in vivo and in vitro [194, 195]. The potential harmfulness of mercury in CVD was first observed in the Kuopio Ischemic Heart Disease Risk Factor (KIHD) study cohort [26]. Several follow-up studies on KIHD study cohort confirmed their observations [196, 197]. In agreement with KIHD study results, it has been suggested in the European Multicenter Case-Control Study on Antioxidants, Myocardial Infarction, and Cancer of the Breast (EURAMIC) study, that high mercury content may diminish the beneficial effects of fish consumption on cardiovascular health [198]. Likewise, in the Health Professionals Follow-up Study (HPFS), increased cardiovascular risk following mercury exposure among dentists, who have an occupational exposure to mercury vapor via amalgam, was consistent with the results from the KIHD and EURAMIC studies [199]. However, these findings were not supported by prospective studies conducted in Sweden [200]. Unlike the previous studies, this population included women and had too low mercury levels, and the range of mercury may have been too narrow to exert sufficient statistical power to detect an association. Apparent discrepancies might be attributed to methodological limitations which are very common in epidemiological studies. In many studies, there are uncertainties in exposure quantification, outcome ascertainment (e.g., misclassification bias), and a lack of information about exposure to other metals and traditional risk factors that are of confounding effects. 

Current uncertainties in the role of mercury in the development of hypertension and diabetes mellitus could be attributed to limited available data [201, 202], and other shortcomings like differences in study design or exposure assessment, lack of sensitive biomarker, and lack of standard criteria for hypertension and diabetes assessment.

#### 7.4.2. Mechanism of Action

Mercury-induced oxidative damage has been observed both in vivo and in vitro, including myocardial tissues. The mechanisms by which mercury exerts its cardiovascular effects are not fully understood. However, exposure to mercury can lead to oxidative stress induction [203], sulfhydryl groups depletion [204], altered mitochondrial function, and apoptosis [205]. 

Mercury-induced redox imbalance may be caused by either increased reactive oxygen species generation or by reduced antioxidants defense capacity. This is supported by observations that antioxidants, both enzymatic and nonenzymatic, can protect against methylmercury toxicity [194]. However, most information is currently derived from animal experimental models and thus implications for human populations consuming mixed diets can only be speculative at this time. 

Mercury can bind to and thus forming complexes with thiol-containing compounds targeting proteins such as glutathione [206], which plays a critical role in regenerating vitamins C and E from their oxidized byproducts. In addition, glutathione-mercury complexes appear to be the primary form in which mercury is transported and eliminated from the body, further decreasing cellular defenses against oxidation. Furthermore, its high affinity for thiol groups and its ability to bind selenium to form an insoluble complex could reduce antioxidative defenses and promote free radical stress and lipid peroxidation in the human body [26]. This interaction between mercury and selenium may represent one mechanism through which mercury increases the risk of CVD, for instance by reducing the bioavailability of selenium or by impairing the activity of glutathione peroxidase. On the contrary, reciprocal interactions are expected, that is, high selenium levels could protect against excess mercury. However, at present, there is very little evidence from human studies to support the hypothesis.

It has been demonstrated that mercury alters the structural integrity of the mitochondrial inner membrane, resulting in loss of normal cation selectivity [194].

Other possible mechanism by which mercury can promote lipid peroxidation and subsequent atherosclerosis is by inhibiting the activation of NF-*κ*B. Mercury may bind to the sulfhydryl groups present in NF-*κ*B and thus impair the activation of NF-*κ*B and attenuate its effects on gene expression [207]. Mercury has been also shown to suppress NO production in in vitro studies by inhibiting the NF-*κ*B pathway and, in that way, inactivating the expression of inducible nitric oxide synthase (iNOS) gene [208]. iNOS catalyzes the production of NO, which has an important role in the maintenance of vascular regulation and immune system [94]. There is some evidence from in vitro studies that mercury can induce changes in platelet aggregation by binding to the thiol groups present in the platelet membrane [209]. However, the exact role of mercury in CVD-related endothelial, inflammatory, and immune functions warrants further investigation.

#### 7.4.3. Combination of Gene-Environmental-Nutrient Interactions

The cardiovascular effect of mercury at lower exposure levels is still subject to controversy. As in the limited understanding of the mechanisms of mercury toxicity [210], nutritional consideration may often be concurrent with or may be additive to genetic predisposition to mercury exposure [211]. However, more focus was attributed to mercury retention by various organs in efforts to explain nutrient mercury interactions [212].

Even though there is ample evidence on food interaction with mercury metabolism at the physiologic level, less certain are the effects of nutrients that might influence bioavailability, toxicodynamics, and transport to target organs and influence the immunologic, biochemical, or cytologic functional responses to mercury. 

Food-like fish has been implicated in the alteration of mercury metabolism. In terms of macronutrient intakes such as fat intake, a positive correlation between dietary mercury and low-density lipoprotein cholesterol levels was observed [213]. Unsaturated fatty acids were also correlated with mercury exposure in populations frequently consuming seafood and fish [198]. However, evidence for protective or antagonistic effects is often complex and highly dependent on metabolic conditions. Studies on the effects of macronutrients on mercury metabolism are expected to shed some light on possible interactions between different nutrients as they have been shown to modulate toxicokinetics and dynamics of mercury metabolism [214]. 

Micronutrients may modify mercury toxicity due to their antioxidant properties. Certain phytochemicals found in the diet reportedly protect against methylmercury toxicity [215]. Such a role is subject to controversy as some antioxidants were found to enhance mercury toxicity in vitro [216]. It is still unknown if these effects are related to antioxidant/pro-oxidant activity or other aspects of mercury metabolism. Of all trace elements, selenium, because of protective effects observed in animal studies, has received the most attention as a potential protector against methylmercury toxicity in populations consuming seafood [217]. Mercury has a high affinity for selenium, and it readily binds selenium to form insoluble mercury selenide complexes [218]. Through this interaction, mercury could reduce the bioavailability of selenium and impair the activity of glutathione peroxidase, thus promoting lipid peroxidation and, subsequently, atherosclerosis. Nonetheless, the combination of high mercury and low selenium was not associated with higher CVD risk [199]. These observations could have been due to limited number of subjects in stratified analysis.

Environmentally induced changes in gene regulatory mechanisms along with dietary interactions may exacerbate mercury intoxication. Metallothionein protein, rich in sulfhydryl groups, helps in scavenging and reducing the toxic effects of mercury. Metallothionein induction is not only seen with mercury but various other metals like cadmium, zinc, and copper [219]. Toxic effects of mercury also induce a number of stress proteins which include heat shock proteins and glucose-regulated proteins that have also been implicated in cardiovascular pathophysiology [220].

## 8. Conclusions

Detrimental effects of heavy metals on the cardiovascular system have been less well defined. A potential proatherogenic effect even if modest compared to other traditional risk factors would have a significant impact in sensitive population groups. However, some issues need to be taken into consideration before one can draw any definitive conclusions. For example, adjustment for confounding variables has been performed in some studies; however, it does not ensure the independence of their association, as it is not possible to measure every conceivable variable. 

Studies summarized in this review point to the harmful effects of heavy metals exposure on the development of CVD. Heavy metals are suspected of inducing pathophysiological changes relevant to atherogenic events including increased oxidative stress, inflammatory response, and coagulation activity. In addition, there are several suggested biological mechanisms that support this hypothesis. The combination of a susceptible genetic background and dietary elements along with environmental coexposure to heavy metals may also explain some aspects of their cardiovascular effects. 

However, the exact mechanism of CVD induced by heavy metals deserves further investigation either through animal experiments or through molecular and cellular studies. Such study designs are optimal to define the cellular and subcellular mechanisms through which they affect the cardiovascular system. Basic insights from the science of cell and molecular biology coupled with improved analytical capabilities have led to the development of better biomarkers embracing the genetic field. There is also a pressing need for the use of sensitive biomarkers in early detection of low-level exposures from new technologies such as nanotechnology. The genetic mechanisms investigated in these studies may also offer new avenues for risk assessment research. Regarding experimental animal models, doses and exposure should be adjusted to long-term low exposure levels that are usually found in human population. Findings based on these studies can lead to the identification of a coherent and consistent biological research pathway for biomarker validation and acceptance into public health practice. Furthermore, large-scale prospective studies with followup on general populations using appropriate biomarkers and cardiovascular endpoints might be recommended to identify the factors that predispose to heavy metals toxicity in CVD.

## Figures and Tables

**Table 1:** Classification of CVD risk factors.

Category	Examples	References
Nonmodifiable risk factors	(i) Advancing age (ii) Male gender (iii) Family history/genotype	(i) [6] (ii) [7] (iii) [8]

Metabolic risk factors	(i) Hypertension (ii) Diabetes mellitus/glucose intolerance (iii) Metabolic syndrome (iv) Hyperlipidemias (v) Obesity/overweight	(i) [9] (ii) [10] (iii) [11] (iv) [12] (v) [13]

Lifestyle risk factors	(i) Smoking (ii) Physical activity (iii) Diet	(i) [14] (ii) [15] (iii) [16, 17]

Novel risk factors	(i) Lipoprotein (a) (ii) Homocysteine (iii) Inflammatory markers (e.g., C-reactive protein) (iv) Prothrombotic factors (e.g., fibrinogen) (v) Trace elements (e.g., selenium, zinc, copper, chromium) (vi) Heavy metals (e.g., arsenic, lead, cadmium, mercury)	(i) [18] (ii) [19] (iii) [20–23] (iv) [24–27]

**Table 2:** Noncardiovascular harmful effects of heavy metals.

Heavy metal	Most affected organs	Chronic health effects	References
Arsenic	(i) Central nervous system (ii) Lungs (iii) Digestive tract (iv) Circulatory system (v) Kidneys	(i) Cancers (ii) Peripheral vascular disease, which in its extreme form leads to gangrenous changes (black foot disease, only reported in Taiwan) (iii) Skin lesions (melanosis, keratosis) (iv) Hearing loss (v) Reproductive toxicity (vi) Hematologic disorders (vii) Neurological diseases (viii) Developmental abnormalities and neurobehavioral disorders	[28]

Lead	(i) Central nervous system (ii) Erythropoiesis (iii) Kidneys (iv) Liver	(i) Cancers (ii) Kidney damage (iii) Neurological diseases (iv) Impaired intellectual ability and behavioral problems in children	[29]

Cadmium	(i) Kidneys (ii) Bone (iii) Liver (iv) Lungs	(i) Cancers (ii) Kidney damage (iii) Bronchiolitis, COPD, emphysema, fibrosis (iv) Skeletal damage, first reported from Japan, the itai-itai (ouch-ouch) disease (a combination of osteomalacia and osteoporosis)	[30]

Mercury	(i) Central nervous system (ii) Kidneys (iii) Liver (iv) Lungs	(i) Lung damage (ii) Kidney damage (iii) Neurological diseases (iv) Impaired intellectual ability and behavioral problems in children (v) Metallic mercury is an allergen, which may cause contact eczema (vi) Mercury from amalgam fillings may give rise to oral lichen	[31]
